# Quality of life assessment instruments for adults: a systematic review of population-based studies

**DOI:** 10.1186/s12955-020-01347-7

**Published:** 2020-06-30

**Authors:** Nila Patrícia Freire Pequeno, Natália Louise de Araújo Cabral, Dirce Maria Marchioni, Severina Carla Vieira Cunha Lima, Clélia de Oliveira Lyra

**Affiliations:** 1 Postgraduate Program in Public Health at the Federal University of Rio Grande do Norte, Avenida Senador Salgado Filho, 1787, Lagoa Nova, Natal, RN Brazil; 2 Department of Nutrition at the Federal University of Rio Grande do Norte, Avenida Senador Salgado Filho, 3000, Lagoa Nova, Natal, RN CEP 59078-970 Brazil; 3 Department of Nutrition of the School of Public Health at the University of São Paulo, Av. Dr. Arnaldo, 715, Cerqueira César, São Paulo, SP Brazil

**Keywords:** Quality of life, Health-related quality of life, Population surveys, Systematic review

## Abstract

**Background:**

Against a backdrop of population aging and improving survival rates for chronic noncommunicable diseases (CNCD), researchers are placing growing emphasis on health-related quality of life (HRQoL). The aim of this study was to identify the QoL assessment instruments used in population-based studies with adults conducted around the world.

**Methods:**

A systematic review of original research published in all languages between 2008 and 2018 was conducted. Systematic reviews and meta-analyses were excluded.

**Results:**

Sixty-three articles (38.1% conducted in the Americas) fitted the eligibility criteria. Based on the AHRQ checklist for cross-sectional studies and the Newcastle-Ottawa scale for cohort studies, methodological quality was shown to be fair in the majority of studies (55.6%) and good in 44.4%. The country with the highest number of publications was Brazil (20.6%). Twelve types of generic instruments and 11 specific instruments were identified. The generic instrument SF-36 was the most frequently used measure (33.3% of studies). In-home interviewing was exclusively used by 47.6% of the studies, while 39 studies (61.9%) reported the use of self-administered questionnaires. Over two-thirds of the studies (34.9%) used questionnaires to investigate the association between chronic diseases and/or associated factors.

**Conclusions:**

It was concluded that the wide range of instruments and modes of questionnaire administration used by the studies may hinder comparisons between population groups with the same characteristics or needs. There is a lack of research on QoL and the factors affecting productive capacity. Studies of QoL in older persons should focus not only on the effects of disease and treatment, but also on the determinants of active aging and actions designed to promote it. Further research is recommended to determine which QoL instruments are best suited for population-based studies.

## Background

Quality of life (QoL) is a multidimensional concept that refers to an “individual’s perception of their position in life in the context of the culture and value systems in which they live and in relation to their goals, expectations, and standards” and is affected by a person’s physical health and psychological state [1]. It can therefore be assumed that the assessment of QoL should consider aspects of physical health, psychological state, level of autonomy, social relationships, beliefs, and relationship to salient features of the environment [2].

Globally, the proportion of older persons is growing and survival rates for chronic noncommunicable diseases (CNCD) are improving. Quality of Life is clinically related to several CNCD, the most common of which are cardiovascular disease, diabetes, hypertension, dyslipidemia and obesity. Clinical and epidemiological research has therefore tended to emphasize the physical health aspects of QoL, focusing on individuals’ perceptions of their living conditions in face of illness and their capacity to lead a meaningful life [3].

Given the complex nature of the concept, the assessment of QoL is a complex undertaking requiring multiple measures to capture subjectivity and multidimensionality. Various instruments have been developed to measure the above domains, adding the subjective parameters considered necessary for a comprehensive assessment of QoL [4].

The most widely used instruments are either generic, which provide an overall assessment of the impacts of health status, or specific, designed to measure particular aspects of QoL, such as QoL related to oral health, visual function, cancer, HIV, etc. [5].

The body of literature on QoL has steadily grown over recent years, spurred by the promotion of research and the cross-cultural adaptation and validation of assessment instruments in different languages [5]. However, limited information exists on the most commonly used instruments against the backdrop of current demographic and epidemiological trends. In light of the above, the aim of this study was to identify QoL assessment instruments used in population-based studies conducted with adults.

## Methodology

A systematic literature review was conducted of studies around the world looking at population-based QoL surveys involving adults. The review followed the recommendations contained in the *Preferred reporting items for systematic review and meta-analyses protocols (PRISMA-P) statement 2015* [6], which provides guidelines for the dissemination of systematic reviews and meta-analyses in healthcare. The review was registered in PROSPERO International prospective register of systematic reviews (registration number CRD42018101934) and is available at the following link: https://www.crd.york.ac.uk/PROSPERO/display_record.php? RecordID = 101,934.

### Search strategy

A search for original articles published between 2008 and 2018 was conducted on the PubMed, Scopus and LILACS electronic databases. A search strategy was performed using the terms MeSH ‘quality of life’, ‘quality of life’, ‘life scales’, ‘HRQOL’, ‘adult’, ‘adults’, ‘elderly’, ‘elderly’, cross-sectional studies “,” Surveys “,” national survey “and” national survey” for each analyzed database (Additional Files 1, 2 and 3). The searches were performed during the period June to August 2018 and were limited to articles published between January 2008 and August 2018 in any language.

### Inclusion and exclusion criteria

Articles were considered eligible if they met the following inclusion criteria: 1) Population-based studies; 2) Conducted with adults; 3) Surveys undertaken in the last 10 years (2008 to 2018); and 4) Studies involving QoL assessment instruments. Systematic reviews and meta-analyses were excluded.

### Synthesis and comparison of results

Initially, two examiners (NP and NC) each carefully read the article titles and abstracts to identify the articles that fitted the eligibility criteria. Where doubts arose regarding inclusion, the entire article was read. Each examiner then read the selected articles in their entirety and filled in a data extraction form prepared by the research team containing questions based on the Newcastle Ottawa Scale (NOS) quality assessment form and Agency for Healthcare Research and Quality (AHRQ) checklist for assessing the quality of studies and other information on the articles: 1) Study characteristics: author(s); year of publication; study locality (country and continent); methodological quality score; and target population (number of participants and age group). 2) Survey characteristics: study name; year; applicability; study purpose; QoL assessment instrument used; mode of questionnaire administration (face-to-face, email, mail); and whether the examiner/interviewers received training. Disagreements between the examiners were clarified via discussion. Where disagreement persisted, a third examiner was invited to make the final decision.

### Assessment of the methodological quality of the studies

The methodological quality of studies was assessed using the AHRQ checklist for cross-sectional studies and the NOS quality assessment form for cohort studies.

The AHRQ checklist contains 11 items with options “Yes”, “No”, or “Unclear”. Items answered “No” or “Unclear” are scored “0” and those answered “Yes” are given “1” [5]. Articles are classified into the following categories based on the total score: good - 8-11, fair - 4-7, and poor - 0-3. The NOS consists of eight items organized into three broad domains: selection of study groups, comparability of the groups, and ascertainment of exposure. Each item is allocated one or no star and articles are classified as “good”, “adequate”, or “poor” according to the number of stars obtained.

To aid presentation, the scores of the articles assessed using the NOS were translated to the AHRQ standards good, fair, and poor using the conversion thresholds developed by the AHRQ [7].

### Criteria used to evaluate the QoL assessment methodology

The following indicators were used to evaluate the QoL assessment methodology used in the studies: 1) Type of QoL assessment instrument; 2) Interviewer training; 3) Mode of questionnaire administration; and 4) Objective of QoL assessment.

## Results

**Fig. 1:**
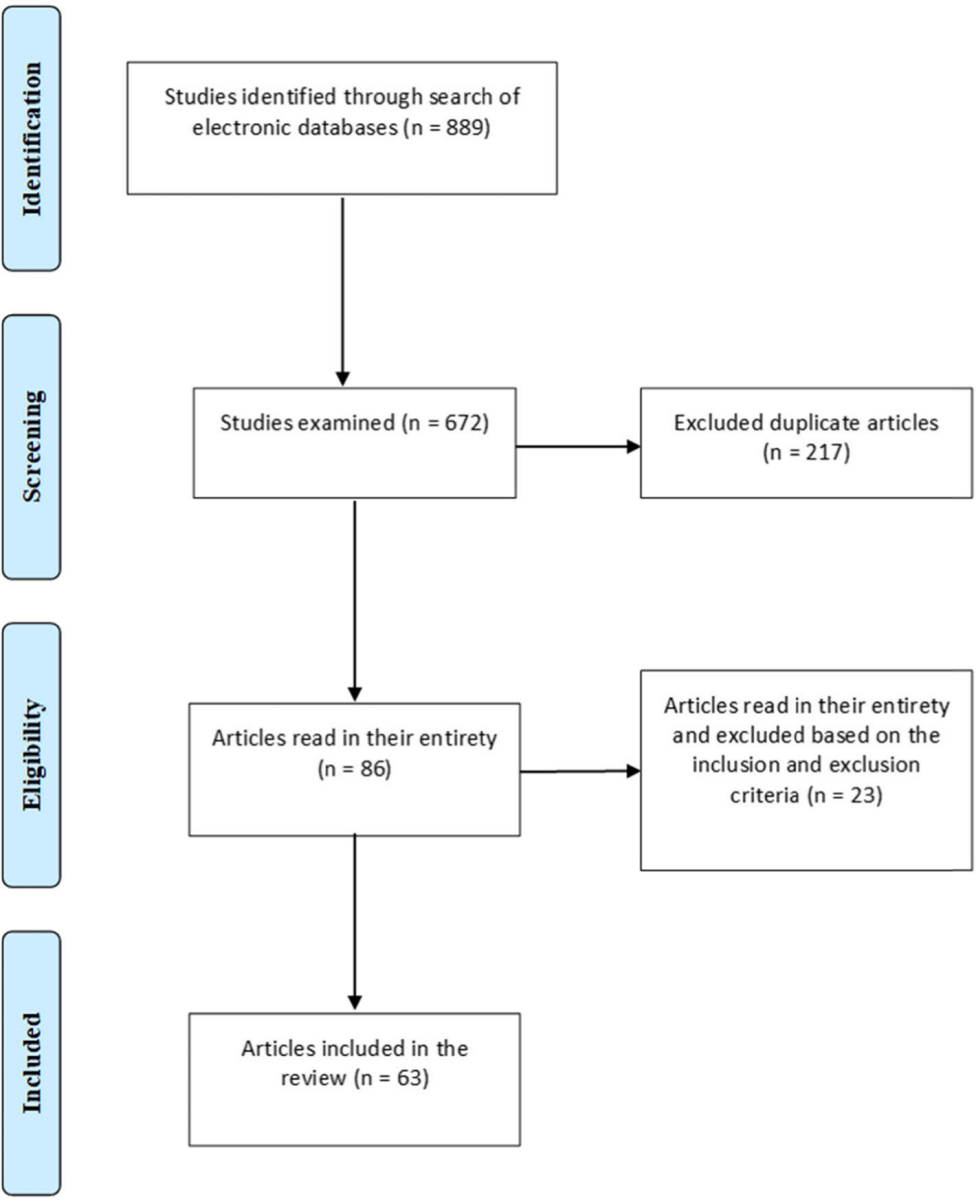
Flowchart of the article selection process

The searches found 889 articles, 217 of which were duplicate and therefore excluded, resulting in the selection of 672 abstracts. After reading the titles and abstracts 566 articles were excluded. After reading the 86 remaining articles in their entirety, 63 were shown to fit the eligibility criteria and included in the final review. The survey is displayed graphically according to the PRISMA diagram (Fig. 1) and was conducted according to the PRISMA guidelines for reporting in systematic reviews (Additional file 4).

### Methodological quality and study characteristics

**Fig. 2:**
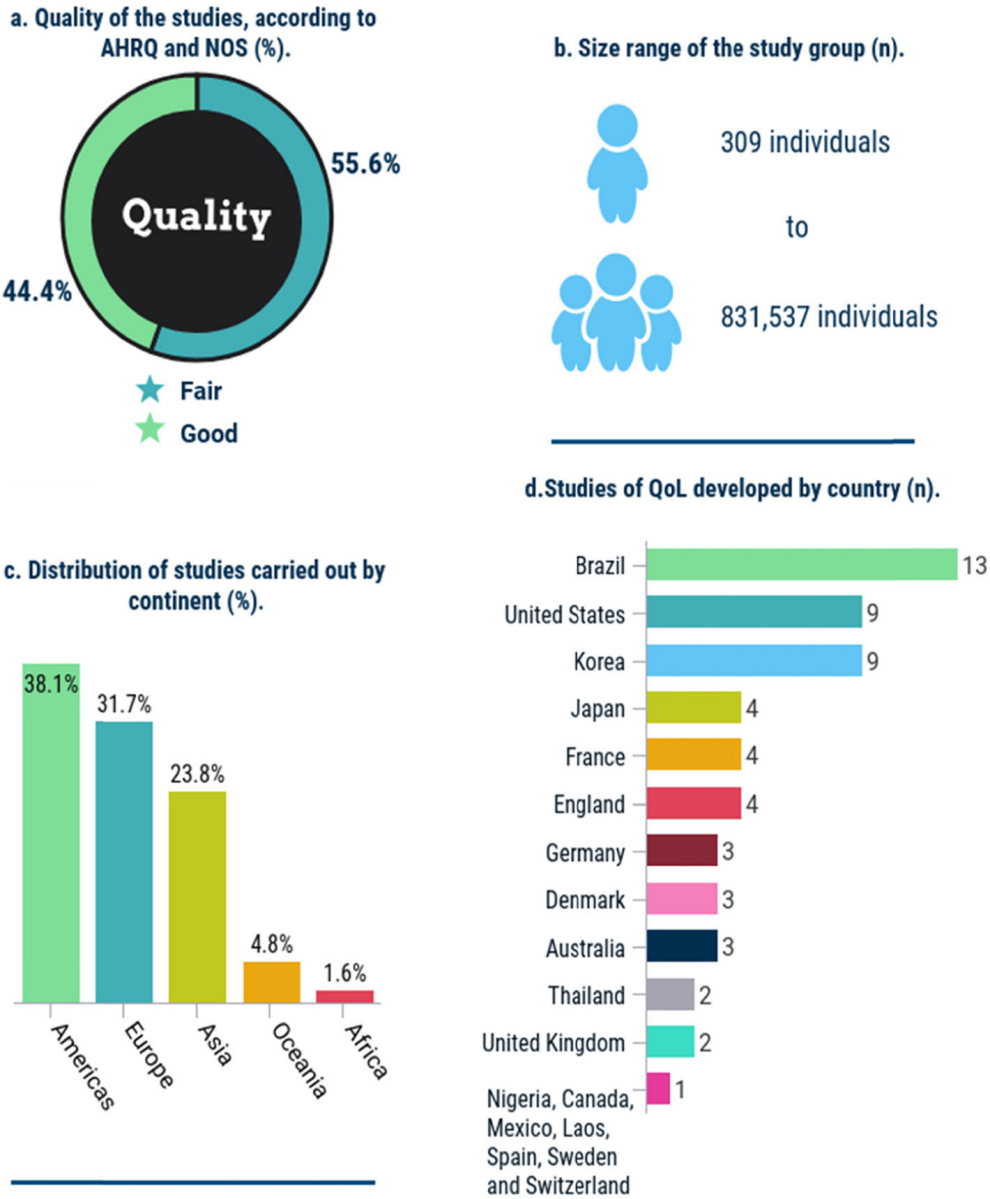
Methodological quality and study characteristics. References: a. Fair quality: [8–42]. Good quality: [43–70]. b [28]. and [57]. c. Americas [8–16, 28–34, 43–49, 58], Europe [17–23, 35–38, 50–52, 57, 59–63], Asia [24–26, 39–41, 53–55, 64–69], Oceania [27, 56, 70] and Africa [42]. d. Brazil [8, 11, 13–16, 29, 33, 34, 43–45, 49], United States [9, 10, 12, 30–32, 46, 47, 58], Korea [26, 40, 41, 55, 64–68], Japan [24, 25, 53, 69], France [18, 61–63], England [19–21, 35], Germany [17, 23, 59], Denmark [37, 52, 60], Australia [27, 56, 70], Thailand [39, 54], United Kingdom [22, 57], Nigeria [42], Canada [28], Mexico [48], Laos [39], Spain [38], Sweden [36], and Switzerland [51]

Figure 2 shows the results for methodological quality and study characteristics (Fig. 2). Further details are shown in supplementary Table 1 (see additional file 5).

### Survey characteristics and instruments used to assess quality of life

The surveys that produced the largest number of publications were the *Korea National Health and Nutrition Examination Survey* – KNHANES (Korea) [26, 41, 55, 64–67], with seven articles. The surveys assessed by this study are listed in Table 1.

**Table 1:** Population surveys that investigated quality of life conducted in countries from Africa, the Americas, Asia, Europe, and Oceania between 2008 and 2018

Country	Survey	References
Korea	*Korea National Health and Nutrition Examination Survey* – KNHANES	[26, 41, 55, 64–67]
	*Korean Community Health Survey* – KCHS	[40]
	The *Korean National Cancer Center*	[68]
Brazil	*Inquérito de Saúde do Município de São Paulo* - ISA Capital-SP	[8, 45]
	*Brazilian Osteoporosis Study* – BRAZOS	[44]
	*Epi Floripa Idoso*	[43]
	*Inquérito da Universidade do Vale do Rio dos Sinos*	[34]
	*Inquérito da Universidade Estadual de Montes Claros*	[11]
	*Inquérito da Universidade Federal de Minas Gerais*	[15]
	*Inquérito de Saúde do Município de Campinas* - ISA Campinas-SP	[13, 49]
	*Brazil National Health and Wellness Survey* – NWHS	[14]
	Pesquisa Dimensões Sociais das Desigualdades – PDSD	[33]
	*Research of the Social Dimensions of Inequalities*	[16]
	*National Survey of Oral Health Brazilian population databases* – SB Brazil	[29]
Denmark	*Danish Breast Cancer Cooperative Group* – DBCG	[52, 60]
	*Survey conducted by the University of Copenhagen*	[37]
England	*English General Practice Patient Survey-* GPPS	[20]
	*Health Survey for England - HSE*	[19, 35]
	*National Survey of Health and Development* – NSHD	[21]
United Kingdom	*English General Practice Patient Survey-* GPPS	[57]
	*Adult Dental Health Survey* – ADHS	[22]
US	*National Health and Nutrition Examination Survey* - NHANES	[46, 58]
	*National Survey of Women Veterans* – NSWV	[9, 47]
	*Nationwide Survey of Female Sexual Health*	[32]
	*Medical Expenditure Panel Survey* – MEPS	[12]
	*National Health and Wellness Survey* – NHWS	[30]
	*National survey of the employment concerns of adults living with multiple sclerosis* – NMSS	[10]
	*Porter Novelli’s 2010 HealthStyles*	[31]
Japan	*Japan National Health and Wellness Survey* – NHWS	[24, 69]
	*The Nationwide Survey of Acute Stroke Care Capacity for Proper Designation of Comprehensive Stroke Center in Japan (J-ASPECT) Study*	[53]
	*Korean Epidemiological Catchment Area* – KECA-R	[25]
Germany	*PSO Health*	[17, 23]
	*Cardiovascular disease, Living and Ageing in Halle –* CARLA	[59]
	*Dortmund Health Study* – DHS	[59]
	*Germany National Health Interview and Examination Survey* – GNHIES 98	[59]
	*Cooperative Health Research in the Region of Augsburg Survey* – KORA S4	[59]
	*Study of Health in Pommerania* – SHIP 0	[59]
Australia	*Australian National Survey of Psychosis*	[70]
	*II National Survey of Mental Health and Well-Being* – HSE	[27]
	*Health Study and the National Survey of Adult Oral Health* – NSAOH	[56]
	*Defense Deployed Solomon Islands (SI) Health Study*	[56]
Spain	CadeViMa-Spain	[38]
France	*French Renal Epidemiology and Information Network* and *CRISTAL database*	[18]
	*French Decennial Health Survey*	[62]
	Nationwide survey of members of the French patients’ society Association Francois Aupetit *[AFA]*	[61]
	VESPA 1 and 2 - ANRS	[63]
Mexico	*Integral study of depression among older adults in Mexico City’s/Mexican Institute of Social Security* – IMSS	[48]
Canada	*Manitoba IBD Cohort Study*	[28]
Thailand	*National Health Interview Suvey in Taiwan*	[54]
The Netherlands	*Second Dutch National Survey of General Practice*	[50]
Nigeria	*The Nigerian national blindness and visual impairment survey*	[42]
Sweden	*The Swedish Survey of Living Conditions*	[36]
Switzerland	*“Vivre/Leben/Vivere”*	[51]
Laos and Thailand	*WHO-ThaiHealth*	[39]

Twenty-three different QoL assessment instruments were used in the surveys, 12 of which were generic (Table 2) and 11 specific instruments (Table 3). The most commonly used instruments were the Medical Outcomes Study Short-Form 36 (MOS SF-36), found in 21 publications [8, 13, 15, 16, 18, 21, 30, 33, 36, 45, 48–50, 52–54, 59–63], EuroQol EQ-5D, used by 17 studies [12, 20, 25, 26, 32, 35, 38–41, 51, 55, 57, 64–67], 12-Item Short-Form Health Survey (SF-12), found in 12 articles [9, 11, 12, 14, 24, 27, 30, 32, 47, 59, 63, 69], and Visual Analogue Scale EQ-VAS, used in seven surveys [17, 23, 26, 38, 40, 55, 66].

**Table 2:** Generic quality of life assessment instruments used in population-based surveys 2008–2018

Abbreviated QoL Instrument	References
AQoL-4D	[70]
CASP-16	[43]
EQ-5D	[12, 20, 25, 26, 32, 35, 38–41, 51, 55, 57, 64–67]
EQ-VAS	[17, 23, 26, 38, 40, 55, 66]
CDC-HRQoL-4	[46]
CDC-HRQoL-14	[58]
PROMIS	[31]
QoL scale	[10]
SF-8	[44]
SF-12	[9, 11, 12, 14, 24, 27, 30, 32, 47, 59, 63, 69]
SF-36	[8, 13, 15, 16, 18, 21, 30, 33, 36, 45, 48–50, 52–54, 59–63]
EUROHIS-QoL 8-item	[34]

*AQoL-4D* Assessment of Quality of Life, *CASP-16* Control, Autonomy, Self-realization and Pleasure; *EQ-5D* EuroQol, *EQ-VAS* Visual Analogue Scale, *CDC HRQoL–14* Healthy Days measures, *CDC HRQoL-4* Healthy Days core questions, *PROMIS* Patient-Reported Outcomes Measurement Information System - Global Health Scale, *QoL scale* Quality of life scale, *SF-8* 8-Item Short-Form Health Survey *SF-12* 12-Item Short-Form Health Survey; *SF-36* Medical Outcomes Study Short-Form 36, EUROHIS-QoL 8-item index

**Table 3:** Specific quality of life assessment instruments used in population-based surveys 2008–2018

Abbreviated QoL Instrument	References
AQLQ-M	[19]
CQoLC-K	[68]
DLQI	[17, 23]
EORTC-QLQ-C30	[37, 68]
FLQA-d	[17]
IBDQ	[28]
OHIP-14	[22, 56]
OIDP	[29]
RTQ	[18]
SIBDQ	[61]
Visual Function/QoL	[42]

*AQLQ-M* Asthma Quality-of-Life Questionnaire, *CQoLC-K* Caregiver Quality of Life Index-Cancer Korean version, *DLQI*, Dermatology Life Quality Index, *EORTC-QLQ-C30* The European Organization for Research and Treatment of Cancer Quality of Life Questionnaire Core 30; *FLQA-d* Freiburg Quality of Life Assessment for Dermatitis, *IBDQ* Inflammatory Bowel Disease Questionnaire, *OHIP-14*, Oral Health Impact Profile, *OIDP* The Oral Impacts on Daily Performance, *RTQ* ReTransQol, *SIBDQ* Short Inflammatory Bowel Disease Questionnaire, Visual Function/QoL

With respect to mode of questionnaire administration, 47.6% of surveys (*n* = 30) used in-home interviewer-administered questionnaires [8, 11, 13, 15, 16, 22, 25, 27–29, 32–35, 38–40, 42–46, 48–51, 54, 58, 62, 68]. Seventy-nine point 4 % of studies (*n* = 50) used interviewer-administered questionnaires, conducted either in-home, in health centers, mobile units, or by telephone [8–11, 13, 15–17, 19, 20, 22, 25–29, 32–35, 38–52, 54–58, 60, 62–68, 70]. Of the 50 studies that used interviewer-administered questionnaires, 64.0% (*n* = 32) reported that the interviewers received training [8, 10, 11, 14, 22, 24, 25, 27, 29, 33, 34, 38–40, 42–46, 53, 54, 56–58, 60, 63–66, 68–70]. The remaining 19% of studies (*n* = 12) used email and mail questionnaires and online panels [13, 16, 18, 26, 35, 36, 48, 50, 55, 61, 62, 67].

When an article failed to provide information about questionnaire administration, we referred to previous publications cited in the article describing the survey methodology, making it possible to capture data on the self-administration of questionnaires.

Thirty-nine (61.9%) studies reported using self-administered questionnaires [8, 10, 12, 13, 16–20, 22, 24–27, 30, 35–37, 39–41, 43, 47, 48, 50, 52–55, 57, 59–67] with or without the presence of an interviewer. Questionnaires were read out to the respondent and the answers filled-in by the interviewer in 28.6% (*n* = 18) of the studies [11, 14, 15, 21, 23, 29, 31–34, 42, 46, 49, 51, 56, 58, 68, 70]. Six articles (9.5%) failed to report who filled in the questionnaire [11, 13, 15, 22, 33, 54].

**Fig. 3:**
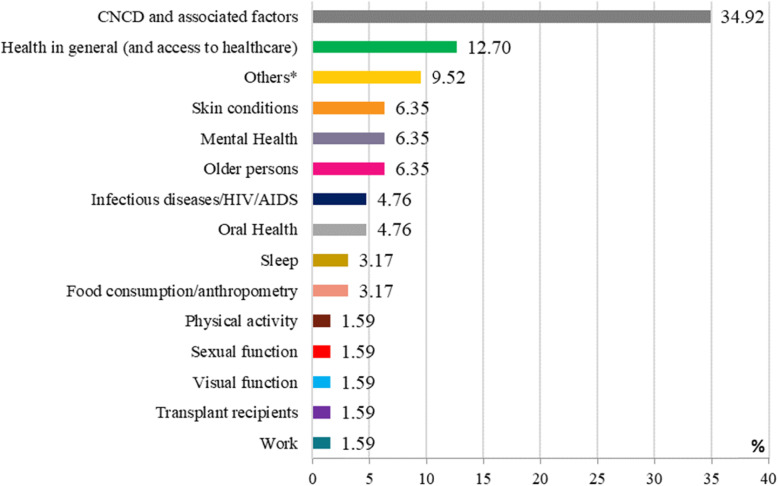
Themes related to quality of life assessment investigated by population-based studies conducted between 2008 and 2018 (*n* = 63 studies). *Others: Comparison of quality of life between ethnic minorities and nontraditional community, informal careers, family members of heavy drinker, social relationships, racial discrimination, and quality of life in general

**Fig. 4:**
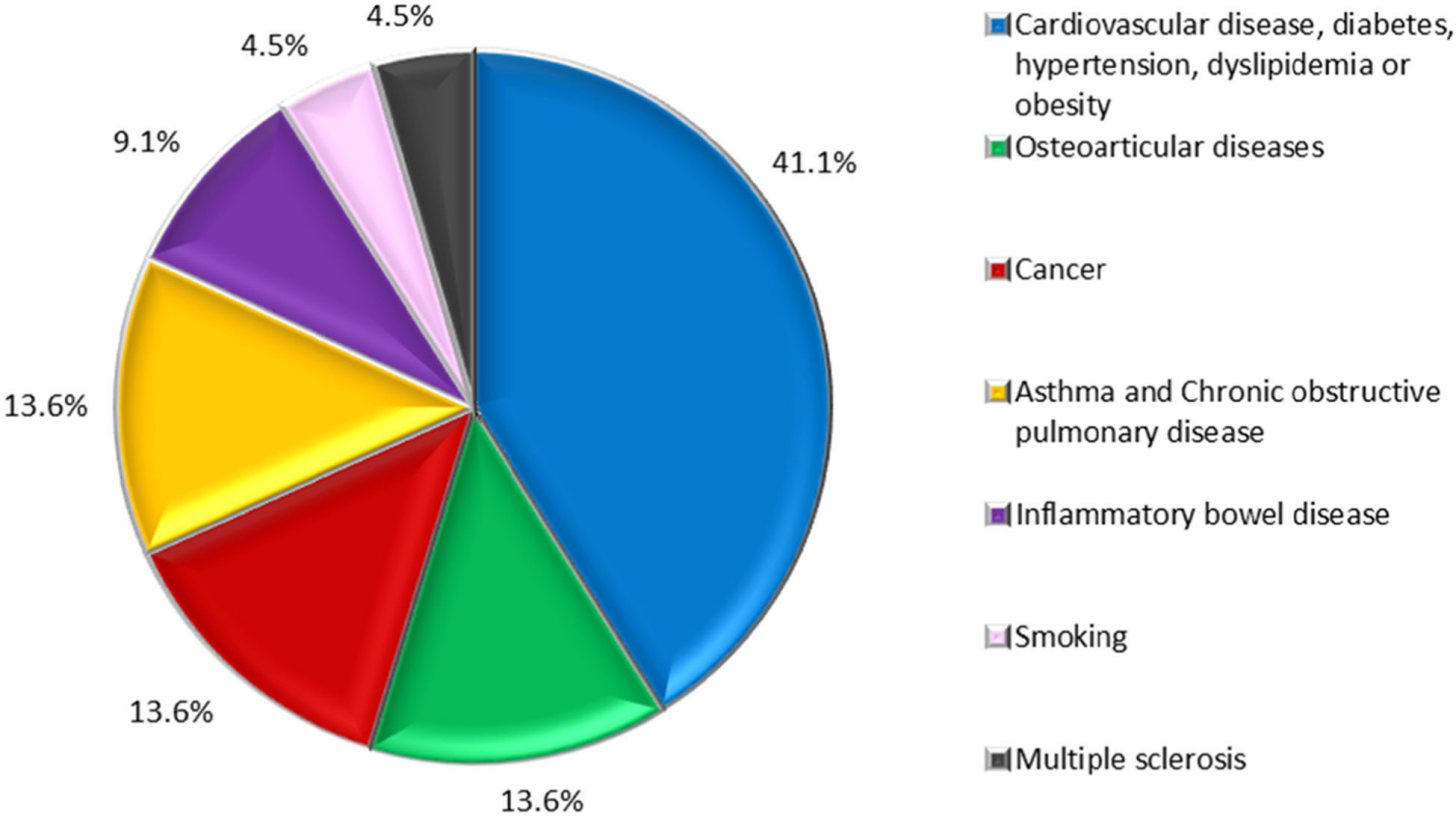
Focus of studies evaluating CNCD and associated factors (*n* = 22 studies)

With regard to the objectives of QoL assessment, 34.92% (*n* = 22) of the studies investigated factors associated with CNCD [10, 11, 15, 19, 26, 28, 30, 31, 37, 40, 41, 52, 55, 57, 59–62, 64–67], while 12.7% (*n* = 8) assessed overall health status or access to healthcare [8, 9, 12, 21, 44, 45, 47, 53] (Fig. 3). Of the 22 studies that investigated the association between QoL and CNCDs, 41,1% addressed cardiovascular disease, diabetes, hypertension, dyslipidemia, and/or obesity [11, 15, 31, 55, 57, 59, 62, 64, 67] (Fig. 4).

With respect to study groups, 13 studies focused exclusively on older persons [8, 21, 22, 27, 29, 38, 43, 45, 47–49, 51, 64], investigating matters such as overall health, oral health, chronic diseases, sleep quality, access to healthcare, successful aging, and social relationships.

## Discussion

The findings show that the majority of surveys opted for generic QoL assessment instruments. This type of instrument, created to provide an objective measure of subjective sensations, has been widely used to assess the various domains of the health status of different populations [11]. Because they are multidimensional, these instruments are widely applicable, allowing researchers to compare QoL between healthy and sick individuals, patients with the same disease, and across different social and cultural backgrounds. However, they are not sensitive to specific aspects of QoL related to a particular morbidity [33].

Instruments used to measure health-related QoL (HRQoL) generally contain questions divided into groups (domains or components) and are designed to assess specific problems that limit health and well-being [11]. The World Health Organization Quality of Life Assessment (WHOQOL), Medical Outcomes Study 36-Item Short Form (SF-36), and 12-Item Short-Form Health Survey (SF-12) are among the most widely used instruments for assessing HRQoL [11].

With respect to administration of QoL instruments, the variety of methods used in the studies illustrates their practicality and ease of use for any study group and situation. The findings show that both generic and specific questionnaires can be administered by health professionals or properly trained third parties. Interviewing is the most commonly used method because it increases response rates and reduces mistakes due to misinterpretation or misunderstanding of questions. The findings show that 38.0% (*n* = 19) of the articles did not report interviewer training. This is concerning, because lack of training and standardization of the interview format, process cost, and presence of an interviewer can influence results [71].

In the case of self-administered questionnaires, understanding of questions and responses rates are influenced by the respondent’s education level. The absence of an interviewer increases the likelihood of misunderstanding and missing data due to missing responses and feelings of anxiety and insecurity experienced by the respondent [71, 72]. Illiterate people are more likely to be excluded from studies if they do not have anyone to assist them [71], which is an important consideration when conducting assessments with study groups with a low level of education [29, 42, 48, 73, 74]. For these groups, face-to-face interview-administered questionnaires are likely to be the best option, as observed in studies from Brazil [29], Mexico [48], and Nigeria [42]. Another bias that can arise from self-administered questionnaires is the tendency of respondents to distort responses in a favorable direction to avoid negative answers. On the other hand, privacy and low cost are potential advantages of this mode of administration [71, 75].

Questionnaires can also be administered by telephone, online panels, or mailed [71, 72]. The findings show that studies used email questionnaires [12, 24] and online questionnaires via online panels [10, 14, 20, 30, 57, 69]. The use of the internet to administer questionnaires, including QoL assessments, has risen considerably. This mode of administration has a number of advantages over pen and paper, including ease of completion, greater integrity, and elimination of data entry errors that can occur when transcribing responses from paper questionnaires. Studies have shown that the ease of use of electronic devices such as laptops, tablets, and smartphones results in greater questionnaire compliance and satisfaction, without compromising the psychometric quality of data, suggesting that electronic questionnaires can generally be considered equivalent to pen-and-paper versions, thus reducing bias, even among respondents who are less tech-savvy [76–78]. However, for online administration of questionnaires to provide results that are equally valid as pen-and-paper administration, each questionnaire should be validated for internet administration [79].

The majority of HRQoL studies related to CNCD focus on correlating the scores of subjective components of QoL with the CNCD and/or its risk factors. Our findings show that the main focus of QoL research was CNCD, notably cardiovascular disease, hypertension, diabetes, dyslipidemia, and/or obesity. The studies evaluated by the present study showed that the presence of these diseases and their risk factors has an impact on quality of life [15, 57, 62, 67].

In this respect, QoL assessment instruments have helped to raise important questions about the QoL of people with CNCD, particularly among adults, given they are the mainstay of a country’s productive capacity. Some of the studies indicated that research should be more focused on the development and review of national strategies for maximizing coordination of care for patients with chronic diseases and reducing the health burden and on the formulation of policies geared towards maintaining physical and mental well-being and improving HRQoL [11, 19, 31]. Collective and individual actions to promote the health of people at risk of CNCD, focusing not only on risk reduction, but also on increasing the chances of improving quality of life, were also highlighted as solutions [15].

Studies showed that QoL scores in the physical and/or mental health domains were lower in individuals with the following CNCD and/or associated factors: diabetes, high blood pressure, obesity, cancer, asthma, osteoarthritis, smoking, excessive alcohol consumption, neurological disorders, long term mental health disorders, chronic back problems, and back pain [11, 15, 19, 26, 40, 57, 60, 65, 66]. In contrast, the highest scores in the physical health domain were associated with the absence of chronic diseases and higher levels of physical activity [11].

The association between combinations of physical and mental health conditions and QoL was investigated by a study conducted by Mujica-Mota et al. [57]. The findings showed that the association between physical health and HRQoL was stronger in the presence of long-term mental health problems, highlighting the importance of addressing these problems, which are often overlooked in patients seeking treatment for physical disorders. According to the authors, integrated approaches to the diagnosis and treatment of long-term health conditions are necessary [57].

Chronic diseases are of long duration and generally slow progression, acting cumulatively to adversely affect health outcomes [31]. With respect to work, consequences of chronic diseases include absenteeism, low productivity and performance, and disability and/or economic inactivity, ultimately affecting the productive capacity of the population [10, 19, 30, 31]. Only a few studies explored the association between chronic diseases and work-related QoL [10, 13, 19, 30]. It is also important to highlight that, besides high treatment costs, poor health outcomes create a financial burden for employers [30, 31]. It is therefore vital to understand patterns of chronic conditions and their effects on QoL and health behaviors to inform interventions to prevent multiple chronic conditions, reduce their burden, and optimize service provision to affected individuals [31].

Declining birth rates, improvements in healthcare, and rising life expectancy have led to a considerable increase in the population of older persons across the globe [31]. Given that studies have reported an association between having a sedentary lifestyle and certain aspects of HRQoL during ageing [80], it is essential to promote healthy lifestyles in order to prevent chronic disease, improve the functional capacity and well-being of older persons, and help maintain autonomy and independence, thus promoting healthy and active ageing [45].

Active ageing is defined by the World Health Organization (WHO) as “the process of optimizing opportunities for health, participation and security in order to enhance quality of life as people age” [81], which is set against the term “health-related quality of life”, a narrow concept focusing on the effects of illness and treatment on quality of life [82]. In this sense, researchers have shown a growing interest in the assessment of QoL in older persons to inform policies to prevent chronic health conditions, prolong life, provide necessary social support, and promote active aging [38]. However, if aging is to be a positive experience, longer life should be accompanied by opportunities for health, participation and security, considering the specific needs, capacities, and preferences of older persons. It is therefore necessary to gain a better understanding of the factors influencing QoL in this group [38].

The findings show that the choice of QoL assessment instrument depends on the type of study. There are no “better or worse” QoL assessment instruments and the decision to use one or another, or a combination of two or more instruments, should depend on the overall purpose of the research [83]. This choice will be influenced by a series of factors, such as the characteristics of the study group and study context [84].

### Implications for health care systems, policy makers and researchers

Our findings show that QoL instruments can help health professionals make informed decisions about disease management. The approach adopted by the instruments assessed in this review and the wide range of aspects of QoL they cover make them valuable tools for monitoring HRQoL. In this respect, they can provide important inputs to support the formulation of policies for improving access to health services and inform the design of health education programs to promote healthy lifestyles and active aging [11, 15, 38]. From a research perspective, we suggest that future population-based studies involving QoL assessment address issues that go beyond the effects of disease or treatment, thus filling the research gaps identified by this review.

### Limitations

This review has its limitations. Despite the widescale use of the World Health Organization Quality of Life Assessment (WHOQOL-100) and its abbreviated version (WHOQOL-BREF) and version for older persons (WHOQOL-OLD) over the last two decades, these instruments were not identified in the studies. One study that used this instrument was preselected in the first stage of the article selection process (reading of titles and abstracts); however, after reading the entire article it was found that it did not meet the eligibility criteria. It is possible that the inclusion criterion *population-based studies* led to the exclusion of other studies that used this instrument. On the other hand, as our findings show that the widespread use of traditional instruments such as the SF-36 and its abbreviated version SF-12 in health research, coupled with the fact that they are quick and easy to use (and therefore particularly useful for large study groups), were reasons for choosing these instruments in the studies analyzed by this review.

## Conclusion

The key findings of this study were as follows: the most frequently-used QoL assessment instrument was the SF-36; the preferred questionnaire administration methods were face-to-face and in-home interviewing with the presence of a trained interviewer; and the main focus of QoL studies was CNCD. There was also a lack of studies of work-related QoL and of positive experiences that promote and enhance the health, participation, and safety of older persons.

It is also important to highlight that while the use of such a wide range of instruments and modes of questionnaire administration may serve to address the specificities of particular study groups, it can hinder comparison between population groups with similar characteristics or needs, thus jeopardizing the validity, statistical reliability and, ultimately, the quality of findings.

This work brings to light important issues that should be addressed by future research aimed at investigating preferences for QoL assessment instruments and determining which instruments are best suited to population-based studies.

## Supplementary information

**Additional file 1.** PUBMED.

**Additional file 2.** SCOPUS

**Additional file 3.** LILACS

**Additional file 4.** PRISMA 2009 Checklist

**Additional file 5.** Suppl Table 1. Characteristics of the studies and population-based surveys of quality of life 2008–2018.

## Data Availability

All data generated or analyzed during this study are included in this published article and its Additional files.

## Abbreviations

AHRQ: Agency for Healthcare Research and Quality
CNCD: Chronic noncommunicable diseases
EQ-5D: EuroQol
EQ-VAS: Visual Analogue Scale
HIV: Human immune deficiency vírus
HRQoL: Health-related quality of life
LILACS: Latin American and Caribbean Health Sciences Literature
MOS SF-36: Medical Outcomes Study Short-Form 36
NOS: Newcastle Ottawa Scale
PRISMA-P: Preferred Reporting Items for Systematic Reviews and Meta-analyses protocols
PROSPERO: International Prospective Register of Systematic Reviews
Qol: Quality of life
SF-12: 12-Item Short-Form Health Survey
WHO: World Health Organization
WHOQOL: World Health Organization Quality of Life Assessment
WHOQOL-100: World Health Organization Quality of Life instrument
WHOQOL-BREF: World Health Organization Quality of Life Assessment abbreviated version
WHOQOL-OLD: World Health Organization Quality of Life Assessment for Older Adults

## References

[1] Group TW (1998). The World Health Organization quality of life assessment (WHOQOL): development and general psychometric properties. Soc Sci Med.

[2] Vagetti GC, Moreira NB, Barbosa Filho VC, de Oliveira V, Cancian CF, Mazzardo O (2013). Domínios da qualidade de vida associados à percepção de saúde: um estudo com idosas de um programa de atividade física em bairros de baixa renda de Curitiba, Paraná. Brasil Cien Saude Colet.

[3] de Queiroz FA, Pace AE, dos Santos CB (2009). Adaptación Cultural Y Validación Del Instrumento Diabetes – 39 ( D-39 ): Versión Para Brasileños Con Diabetes Mellitus Tipo 2 - Fase 1 1 Cross-Cultural Adaptation and Validation of the Instrument Diabetes – 39 ( D-39 ): Brazilian Version for Type 2 Diabet. Rev Latino Am Enferm.

[4] Campolina AG, Dini PS, Ciconelli RM (2011). Impacto da doença crônica na qualidade de vida de idosos da comunidade em São Paulo ( SP , Brasil ) The impact of chronic disease on the quality of life of the elderly in São Paulo (SP, Brazil). Ciência e Saúde Coletiva.

[5] Landeiro G, Pedrozo C, Gomes M, Oliveira E (2011). Revisão sistemática dos estudos sobre qualidade de Vida indexados na base de dados Scielo systematic review of studies on quality of life indexed on the Scielo database. Ciênc saúde coletiva.

[6] Moher D, Shamseer L, Clarke M, Ghersi D, Liberati A, Petticrew M (2015). Preferred reporting items for systematic review and meta-analysis protocols (PRISMA-P) 2015 statement. Syst Rev.

[7] AHRQ. Agency for Healthcare Research and Quality. Newcastle-Ottawa Quality Assessment Form for Cohort Studies. Available at: https://www.ncbi.nlm.nih.gov/books/NBK115843/bin/appe-fm3.pdf.

[8] Lima MG, Barros MB, César CL, Goldbaum M, Carandina L, Ciconelli RM (2009). Health related quality of life among the elderly: a population-based study using SF-36 survey. Cad Saúde Públic.

[9] Cordasco KM, Mengeling MA, Yano EM, Washington DL (2016). Health and health care access of rural women veterans: findings from the National Survey of women veterans. J Rural Health.

[10] Cichy KE (2016). Non-vocational health-related correlates of quality of life for older adults living with multiple sclerosis. J Rehabil.

[11] Noronha DD, Martins AMEdBL, Dias DdS, Silveira MF, Paula AMBD, Haikal DSS (2016). Qualidade de vida relacionada à saúde entre adultos e fatores associados: um estudo de base populacional. Cien Saude Colet.

[12] Stewart ST, Woodward RM, Rosen AB, Cutler DM (2008). The impact of symptoms and impairments on overall health in US National Health Data. Med Care.

[13] Senicato C, Lima MG, Barros MB d. A, Rio (2016). Ser trabalhadora remunerada ou dona de casa associa-se à qualidade de vida relacionada à saúde?. Cad Saúde Pública.

[14] El Khoury AC, Vietri J, Prajapati G (2014). Health-related quality of life in patients with hepatitis C virus infection in Brazil. Rev Panam Salud Publica.

[15] Oliveira-Campos M, Rodrigues-Neto JF, Silveira MF, Neves DMR, Vilhena JM, Oliveira JF, Magalhães JC, Drumond D (2013). Impacto dos fatores de risco para doenças crônicas não transmissíveis na qualidade de vida. Cien Saude Colet.

[16] Pavão A, Ploubidis G, Werneck G, Campos M, Werneck G, Rodrigues Campos M (2012). Racial Discrimination and health in Brazil: evidence from a population-based survey. Ethn Dis.

[17] Langenbruch A, Marc Alexander Radtke B, Arnd Jacobi B, Sandra Purwins B, Haack K, Kristian Reich B (2016). Quality of psoriasis care in Germany: results of the national health care study “‘PsoHealth3’”. Arch Dermatol Res.

[18] Gentile S, Beauger D, Speyer E, Jouve E, Dussol B, Jacquelinet C (2013). Factors associated with health-related quality of life in renal transplant recipients: results of a national survey in France. Health Qual Life Outcomes.

[19] Upton J, Lewis C, Humphreys E, Price D, Walker S (2016). Asthma-specific health-related quality of life of people in Great Britain: A national survey. J Asthma.

[20] Thomas GPAA, Saunders CL, Roland MO, Paddison CAMM (2015). Informal carers’ health-related quality of life and patient experience in primary care: evidence from 195,364 carers in England responding to a national survey. BMC Fam Pract.

[21] Mishra GD, Black S, Stafford M, Cooper R, Kuh D (2014). Childhood and Maternal Effects on Physical Health Related Quality of Life Five Decades Later: The British 1946 Birth Cohort Gita. Schooling C, organizador. PLoS One.

[22] Masood M, Newton T, Bakri NN, Khalid T, Masood Y (2017). The relationship between oral health and oral health related quality of life among elderly people in United Kingdom. J Dent.

[23] Langenbruch A, Radtke M, Augustin M (2012). Quality of psoriasis care from the patients’ perspective – results of the national health care study PsoReal. Eur J Dermatol.

[24] Liu GG, da Costa DBM, Yuan Y, Wagner J-S, L’Italien GJ, Langley P (2012). The burden of illness for patients with viral hepatitis C: evidence from a National Survey in Japan. Value Health.

[25] Cho S-J, Hong JP, Hahm B-J, Jeon HJ, Chang SM, Cho MJ (2009). Restless legs syndrome in a community sample of Korean adults: prevalence, impact on quality of life, and association with DSM-IV psychiatric disorders. Sleep..

[26] Kim W, Jin YS, Lee CS, Bin S, Lee Y, Choi KH (2015). Influence of knee pain and low Back pain on the quality of life in adults older than 50 years of age. PM R7.

[27] Parslow RA, Lewis VJ, Nay R (2011). Successful aging: development and testing of a multidimensional model using data from a large sample of older Australians. J Am Geriatr Soc.

[28] Rawsthorne P, Clara I, Graff LA, Bernstein KI, Carr R, Walker JR (2012). The Manitoba inflammatory bowel disease cohort study: a prospective longitudinal evaluation of the use of complementary and alternative medicine services and products. Gut..

[29] Souza JGS, Costa Oliveira BE, Martins AMEDBL, Gabriel J, Souza S, Bárbara (2017). Contextual and individual determinants of oral health-related quality of life in older Brazilians. Qual Life Res.

[30] Dhamane AD, Witt EA, Su J (2016). Associations between COPD severity and work productivity, health-related quality of life, and health care resource use. J Occup Environ Med.

[31] Barile JP, Mitchell SA, William W, Thompson P, Zack MM, Reeve BB, David Cella P, Ashley Wilder Smith P (2015). Patterns of chronic conditions and their associations with behaviors and quality of life, 2010. Prev Chronic Dis Heal Res Pract Policy.

[32] Biddle AK, West SL, D’Aloisio AA, Wheeler SB, Borisov NN, Thorp J (2009). Hypoactive sexual desire disorder in postmenopausal women: quality of life and health burden. Value Health.

[33] Flor LS, Campos MR, Laguardia J, Sorio Flor Rua Dulce L (2013). Qualidade de vida, posição social e grupos ocupacionais no Brasil: evidência de uma pesquisa de base populacional Quality of life, social position and occupational groups in Brazil: evidence from a population-based survey. Rev Bras Epidemiol.

[34] Backes V, Olinto MTA, Henn RL, Cremonese C, Pattussi MP (2011). Associação entre aspectos psicossociais e excesso de peso referido em adultos de um município de médio porte do Sul do Brasil. Cad Saúde Pública.

[35] Anokye NK, Trueman P, Green C, Pavey TG, Taylor, RoAnokye NK, Trueman P (2012). Physical activity and health related quality of life. BMC Public Health.

[36] Lindberg M, Bingefors K, Isacson D (2011). Quality of life, use of topical medications and socio-economic data in hand eczema: A Swedish Nationwide survey. Acta Derm Venereol.

[37] Madsen UR, Groenvold M, Petersen MA, Johnsen AT, Riis MU, Groenvold @bullet Mogens (2015). Comparing three different approaches to the measurement of needs concerning fatigue in patients with advanced cancer. Qual Life Res.

[38] Fernandez-Mayoralas G, Giraldez-Garcia C, Forjaz MJ, Rojo-Perez F, Martinez-Martin P, Prieto-Flores M-E (2012). Design, measures and sample characteristics of the CadeViMa-Spain survey on quality of life in community-dwelling older adults. Int Psychogeriatrics C Int Psychogeriatr Assoc.

[39] Jintana J, Surasak C, Waleewong Orratai SL, Sengngam Khanpaseuth DD, Thamarangsi T (2017). The impact of heavy drinkers on others’ health and well-being in Lao PDR and Thailand. J Subst Use ISSN.

[40] Chung J, Han C (2017). Health related quality of life in relation to asthma – data from a cross sectional study. J Asthma.

[41] Kim Y, Cho W-K (2014). Factors associated with successful smoking cessation in Korean adult males: findings from a National Survey. Iran J Public Health.

[42] Tran HM, Mahdi AM, Sivasubramaniam S, Gudlavalleti MVS, Gilbert CE, Shah SP (2011). Quality of life and visual function in Nigeria: findings from the National Survey of blindness and visual impairment. Br J Ophthalmol.

[43] Marques LP, Schneider IJC, D’Orsi E. Quality of life and its association with work, the internet, participation in groups and physical activity among the elderly from the EpiFloripa survey, Florianópolis, Santa Catarina state, Brazil. Cad Saude Publica. 2016;32(12):e00143615. 10.1590/0102-311X00143615 28001209

[44] Campolina CAG, Gonçalves Campolina A, Pinheiro MM, Ciconelli RM, Bosi FM (2011). Quality of life among the Brazilian adult population using the generic SF-8 questionnaire. Cad Saúde Pública.

[45] Lima, M.G., Barros M.B, César, C.L.G., Goldbaum, M., Carandina, L. and Alves MCGP. Health-related behavior and quality of life among the elderly: a population-based study Comportamentos relacionados a saúde e qualidade de Vida em idosos: um estudo de base populacional. Rev Saúde Pública 2011;45(3):485–493. 10.1590/s0034-89102011000300006 21552754

[46] Chen X, Gelaye B, Williams MA (2014). Sleep characteristics and health-related quality of life among a national sample of American young adults: assessment of possible health disparities. Qual Life Res.

[47] Der-Martirosian C, Cordasco KM, Washington DL (2013). Health-related quality of life and comorbidity among older women veterans in the United States. Qual Life Res.

[48] Gallegos-Carrillo K, Mudgal J, Sánchez-García S, Wagner FA, Gallo JJ, Salmerón J, García-Peña C (2009). Social networks and health-related quality of life: a population based study among older adults. Salud Publica Mex.

[49] Lima MG. Barros, M.B.d.A. and Alves MCGP, Rio. Sllep Duration And Health Status Self-Assessment in the elderly 2012;28(9):1674–1684. 10.1590/s0102-311x2012000900007 23033183

[50] Hoopman R, Terwee CB, Devillé W, Knol DL, Aaronson NK (2009). Evaluation of the psychometric properties of the SF-36 health survey for use among Turkish and Moroccan ethnic minority populations in the Netherlands. Qual Life Res.

[51] Luthy C, Cedraschi C, Allaz A-F, Herrmann R, Ludwig C, François @bullet (2015). Health status and quality of life: results from a national survey in a community-dwelling sample of elderly people. Qual Life Res.

[52] Peuckmann V, Ekholm O, Sjøgren P, Rasmussen NK, Christiansen P, Møller S (2009). Health care utilisation and characteristics of long-term breast cancer survivors: Nationwide survey in Denmark. Eur J Cancer.

[53] Nishimura K, Nakamura F, Takegami M, Fukuhara S, Nakagawara J, Ogasawara K, Ono J, Shiokawa Y, Miyachi S, Nagata I, Toyoda K, Matsuda S, Kataoka H (2014). Cross-sectional survey of workload and burnout among Japanese physicians working in stroke care. Circ Cardiovasc Qual Outcomes.

[54] Huang C-J, Hu H-T, Fan Y-C, Liao Y-M, Tsai P-S (2010). Associations of breakfast skipping with obesity and health-related quality of life: evidence from a national survey in Taiwan. Int J Obes.

[55] Kim H-J, Lee J-WJ-Y, Kim T-J, Lee J-WJ-Y (2015). Association between serum vitamin D status and health-related quality of life (HRQOL) in an older Korean population with radiographic knee osteoarthritis: data from the Korean national health and nutrition examination survey (2010–2011). Health Qual Life Outcomes.

[56] Crocombe L, Mahoney G, Spencer A, Waller M (2013). Will improving access to dental care improve oral health-related quality of life?. Aust Dent J.

[57] Mujica-Mota RE, Roberts M, Abel G, Elliott M, Lyratzopoulos G, Roland M (2015). Common patterns of morbidity and multi-morbidity and their impact on health-related quality of life: evidence from a national survey. Qual Life Res.

[58] Helmick CG, Lee-Han H, Hirsch SC, Baird TL, Bartlett CL (2014). Prevalence of psoriasis among adults in the U.S: National Health and nutrition examination surveys. Am J Prev Med.

[59] Schunk M, Reitmeir P, Schipf S, Völzke H, Meisinger C, Ladwig K-H (2015). Health-related quality of life in women and men with type 2 diabetes: a comparison across treatment groups. J Diabetes Complicat.

[60] Peuckmann V, Ekholm O, Rasmussen NK, Groenvold M, Christiansen P, Møller S (2009). Chronic pain and other sequelae in long-term breast cancer survivors: Nationwide survey in Denmark. Eur J Pain.

[61] Williet N, Sarter H, Gower-Rousseau C, Adrianjafy C, Olympie A, Buisson A (2017). Patient-reported outcomes in a French Nationwide survey of inflammatory bowel disease patients. J Crohn's Colitis.

[62] Audureau E, Pouchot J, Coste J (2016). Gender-related differential effects of obesity on health-related quality of life via obesity-related comorbidities. Circ Cardiovasc Qual Outcomes.

[63] Douab T, Marcellin F, Vilotitch A, Protopopescu C, Préau M, Suzan-Monti M (2014). Health-related quality of life of people living with HIV followed up in hospitals in France: comparing trends and correlates between 2003 and 2011 (ANRS-VESPA and VESPA2 national surveys). AIDS Care.

[64] Kim M, Kim J, Won CW (2018). Association between involuntary weight loss with low muscle mass and health-related quality of life in community-dwelling older adults: Nationwide surveys (KNHANES 2008–2011). Exp Gerontol.

[65] Park J-H, Hong J-Y, Han K, Suh S-W, Park S-Y, Yang J-H (2017). Prevalence of symptomatic hip, knee, and spine osteoarthritis nationwide health survey analysis of an elderly Korean population. Medicine (Baltimore).

[66] Hong J-Y, Han K, Shin D-H, Chun EM (2016). Quality of life analysis and smoking correlation in symptomatic spine osteoarthritis: A Nationwide health survey analysis of an elderly population with EQ-5D. PLoS One.

[67] Park SJ, Ahn S, Park KH (2016). Burden of visual impairment and chronic diseases. JAMA Ophthalmol.

[68] Sanson-Fisher RW, Wook Shin D, Cho J, Park J-H (2013). Prevalence and predictors of anxiety and depression among family caregivers of cancer patients: a nationwide survey of patient–family caregiver dyads in Korea. Support Care Cancer.

[69] Vietri J, Otsubo T, Montgomery W, Tsuji T, Harada E (2015). Association between pain severity, depression severity, and use of health care services in Japan: results of a nationwide survey. Neuropsychiatr Dis Treat.

[70] Neil AL, Carr VJ, Mackinnon A, Foley DL, Morgan VA (2018). Health-related quality of life in people living with psychotic illness, and factors associated with its variation. Value Health.

[71] Silqueira SM de F. O questionário genérico SF-36 como instrumento de mensuração da qualidade de vida relacionada a saúde de pacientes hipertensos [Internet]. [Ribeirão Preto]: Biblioteca Digital de Teses e Dissertações da Universidade de São Paulo; 2005 [citado 11 de janeiro de 2019]. Available at: http://www.teses.usp.br/teses/disponiveis/22/22133/tde-17052007-160822/.

[72] Guyatt GH, Feeny DHPD (1993). Measuring health-related quality of life. Ann Intern Med.

[73] Cinoto RW, Berezovsky A, Belfort R, Rios Salomão S (2006). Comparison between self-reported quality of vision and visual acuity in a low-income elderly population in the city of São Paulo. Arq Bras Oftalmol.

[74] Nascimento Cruz L, Oliveira MR, Alves Camey S, Hoffmann JF, Bagattini ÂM, Fleck MP de A (2013). Health-related quality of life in Brazil: normative data for the SF-36 in a general population sample in the south of the country. Cien Saude Colet.

[75] Almeida-Brasil CC, Silveira MR, Silva KR, Lima MG, Cardoso CL, Faria CDC de M (2017). Qualidade de vida e características associadas: aplicação do WHOQOL-BREF no contexto da Atenção Primária à Saúde. Cien Saude Colet.

[76] Muehlhausen W, Doll H, Quadri N, Fordham B, O’donohoe P, Dogar N (2015). Equivalence of electronic and paper administration of patient-reported outcome measures: a systematic review and meta-analysis of studies conducted between 2007 and 2013. Health Qual Life Outcomes.

[77] Larsen Rasmussen S, Rejnmark L, Ebbehøj E, Feldt-Rasmussen U, Krogh Rasmussen Å, Bue Bjorner J (2016). High level of agreement between electronic and paper mode of Administration of a Thyroid-Specific Patient-Reported Outcome. ThyPRO Eur Thyroid J.

[78] Broering JM, Paciorek A, Carroll PR, Leslie, Wilson S, Litwin MS (2014). Measurement equivalence using a mixed-mode approach to administer health-related quality of life instruments. Qual Life Res.

[79] Wu R, Thorpe K, Ross H, Micevski V, Marquez C, Straus S (2009). Comparing Administration of Questionnaires via the internet to pen-and-paper in patients with heart failure: randomized controlled trial. J Med Internet Res.

[80] Kim Y, Lee E (2019). The association between elderly people’s sedentary behaviors and their health-related quality of life: focusing on comparing the young-old and the old-old. Health Qual Life Outcomes.

[81] World Health Organization. Active Ageing: A Policy Framework: World Heal Organ; 2002. Available at: https://apps.who.int/iris/bitstream/handle/10665/67215/WHO_NMH_NPH_02.8.pdf;jsessionid=192A324CEE72EF90EAD6E71519F64B72?sequence=1. 12040973

[82] Ferrans C, Zerwic J, Wilbur J, Larson J (2005). Conceptual model of health-related quality of life. J Nurs Scholarsh.

[83] Coons S, Rao S, Keininger DL, Hays R (2000). A comparative review of generic quality-of-life instruments. Pharmacoeconomics..

[84] Gomes JRAA, Hamann EM, Gutierrez MMU. Aplicação do WHOQOL-BREF em segmento da comunidade como subsídio para ações de promoção da saúde. Rev Bras Epidemiol. 2014:495–516. 10.1590/1809-4503201400020016eng 24918419

