# Supplementary material for: Quality of life assessment instruments for adults: a systematic review of population-based studies
Source: Health Qual Life Outcomes. 2020 Jun 30;18:208. doi: 10.1186/s12955-020-01347-7 (PMC7329518; doi:10.1186/s12955-020-01347-7)
Supplement: Supplementary file 2 — Additional file 2. SCOPUS [file 12955_2020_1347_MOESM2_ESM.docx]

**SCOPUS**

TITLE-ABS-KEY ("Quality of life” OR “Life Quality” OR “Scales of life” OR “HRQoL”) AND TITLE-ABS-KEY ("adult” OR “adults” OR "aged” OR “elderly”) AND TITLE-ABS-KEY ("cross-sectional studies” OR “surveys”) AND TITLE-ABS-KEY ("nationwide survey” OR “national survey")
