# Supplementary material for: Quality of life assessment instruments for adults: a systematic review of population-based studies
Source: Health Qual Life Outcomes. 2020 Jun 30;18:208. doi: 10.1186/s12955-020-01347-7 (PMC7329518; doi:10.1186/s12955-020-01347-7)
Supplement: Supplementary file 3 — Additional file 3. LILACS [file 12955_2020_1347_MOESM3_ESM.docx]

**LILACS**

(“*Qualidade de vida*” OR “*escalas de vida*” OR “HRQOL”) AND (“*adultos*” OR “*idosos*”) AND (“*estudos seccionais*” OR “*estudos transversais*” OR “*inquéritos*”) AND (“*nacional*” OR “*populacional*”).
