# Supplementary material for: Quality of life assessment instruments for adults: a systematic review of population-based studies
Source: Health Qual Life Outcomes. 2020 Jun 30;18:208. doi: 10.1186/s12955-020-01347-7 (PMC7329518; doi:10.1186/s12955-020-01347-7)
Supplement: Supplementary file 5 — Additional file 5. Suppl Table 1. Characteristics of the studies and population-based surveys of quality of life 2008–2018. [file 12955_2020_1347_MOESM5_ESM.docx]

Suppl table1. Characteristics of the studies and population-based surveys of quality of life 2008-2018.

| Study characteristics | | | | | | | | Survey characteristics | | | |
| --- | --- | --- | --- | --- | --- | --- | --- | --- | --- | --- | --- |
| Author | **Year** | **Continent** | **Country** | **Study design** | **n** | **Age (Years)** | **Methodological quality(score)** | **Survey** | **Quality of life (QoL) Questionnaire used** | **Applicability of QoL Questionnaire** | **Questionnaire mode of administration** |
| Tran et al.(70) | 2011 | Africa | Nigeria | Cross-sectional | 2,076 | >=40 | 7 (fair) | The Nigerian national blindness and visual impairment survey. | Visual Function/QoL | Visual function | In-home interviewing with interviewer-administered questionnaire |
| Souza et al.(8) | 2017 | Americas | Brazil | Cross-sectional | 7,619 | 65-74 | 5 (fair) | National Survey of Oral Health Brazilian population databases (SB Brazil, 2010) | OIDP | Oral health | In-home interviewing with interviewer-administered questionnaire |
| Marques et al(9) | 2016 | Americas | Brazil | Cross-sectional | 1,131 | >60 | 10 (good) | *Epi Floripa Idoso* (study the health and living conditions of adult and the elderly population in Florianópolis)  (2009-2010) (2013-2014) | CASP-16 | Quality of life of older persons | In-home interviewing with self-administered questionnaire |
| Noronha et al(20) | 2016 | Americas | Brazil | Cross-sectional | 841 | 35-44 | 6 (fair) | Inquérito da Universidade Estadual de Montes Claros | SF-12 | Factors associated with chronic diseases | In-home interviewing; who filled in the questionnaire not reported. |
| Senicato et al(25) | 2016 | Americas | Brazil | Cross-sectional | 668* | 18-64 | 7 (fair) | Inquérito de Saúde de Campinas – ISACamp (2008-2009) | SF-36 | Work | In-home interviewing; who filled in the questionnaire not reported. |
| El Khoury et al(26) | 2014 | Americas | Brazil | Cross-sectional | 11,794 | >18 | 7 (fair) | Brazil National Health and Wellness Survey (NWHS) | SF-12 | Infectious diseases | Self-administered online questionnaire or in-home questionnaire. |
| Flor et al(27) | 2013 | Americas | Brazil | Cross-sectional | 12,423 | >20 | 6 (fair) | Pesquisa Dimensões Sociais das Desigualdades (PDSD) | SF-36 | Overall quality of life | In-home interviewing; who filled in the questionnaire not reported. |
| Oliveira-Campos(28) | 2013 | Americas | Brazil | Cross-sectional | 648 | >18 | 6 (fair) | Inquérito da Universidade Federal de Minas Gerais | SF-36 | Factors associated with chronic diseases | In-home interviewing; who filled in the questionnaire not reported. |
| Lima et al(29) | 2012 | Americas | Brazil | Cross-sectional | 1,418 | >60 | 10 (good) | Inquérito de Saúde de Campinas ISACamp (2008-2009) | SF-36 | Sleep quality | In-home interviewing, with self-administered questionnaire |
| Pavão et al(30) | 2012 | Americas | Brazil | Cross-sectional | 3,863 | >=20 | 6 (fair) | Research for Social Dimension of Inequalities (2008) | SF-36  (one general question about health) | Racial discrimination | In-home interviewing, with self-administered questionnaire. |
| Backes et al(31) | 2011 | Americas | Brazil | Cross-sectional | 1,100 | >18 | 6 (fair) | Inquérito da Universidade do Vale do Rio dos Sinos | EUROHIS-QoL 8-item index | Psychosocial aspects and excess weight | In-home interviewing with interviewer-administered questionnaire |
| Campolina et al(10) | 2011 | Americas | Brazil | Cross-sectional | 2,420 | >40 | 8 (good) | Brazilian Osteoporosis Study (BRAZOS) | SF-8 | Health in general | In-home interviewing with interviewer-administered questionnaire |
| Lima et al(11) | 2011 | Americas | Brazil | Cross-sectional | 1,958 | >60 | 8 (good) | Inquérito de Saúde do Município de São Paulo - ISA Capital-SP (2001-2002). | SF-36 | Health in general | In-home interviewing with interviewer-administered questionnaire |
| Lima et al(12) | 2009 | Americas | Brazil | Cross-sectional | 1,958 | >60 | 6 (fair) | Inquérito de Saúde do Município de São Paulo - ISA Capital-SP (2001-2002). | SF-36 | Health in general | In-home interviewing with interviewer-administered questionnaire |
| Rawsthorne et al(13) | 2012 | Americas | Canada | Cohort | 309 | >18 | 7 (fair) | Manitoba IBD Cohort Study | IBDQ | Assessment of the use of alternative services and products for the treatment of inflammatory bowel disease | In-home interviewing, with self-administered questionnaire. |
| Cordasco et al(14) | 2016 | Americas | US | Cross-sectional | 3,611* | >18 | 7 (fair) | National Survey of Women Veterans (NSWV) | SF-12 | Access to healthcare | Computer-assisted telephone interviewing, with interviewer-administered questionnaire |
| Cichy et al(15) | 2016 | Americas | US | Cross-sectional | 703 | >55 | 5 (fair) | National survey of the employment concerns of adults with multiple sclerosis. (National Multiple Sclerosis Society - NMSS). | QoL scale | Autoimmune diseases (multiple sclerosis) | Self-administered mail or on-line questionnaire; telephone questionnaire |
| Dhamane et al(16) | 2016 | Americas | US | Cross-sectional | 60,479 | >40 | 7 (fair) | National Health and Wellness Survey (NHWS, 2010/2012). | SF-12 (2010-2011)  SF-36 (2012) | Respiratory diseases  (DPOC) | On-line self-administered questionnaire. |
| Barile et al(17) | 2015 | Americas | US | Cross-sectional | 4,184 | >18 | 5 (fair) | Porter Novelli’s 2010 Health Styles | PROMIS | Chronic diseases | Self-administered mail questionnaire |
| Chen et al(18) | 2014 | Americas | US | Cross-sectional | 2,391 | 20-39 | 8 (good) | National Health and Nutrition Examination Survey - NHANES (2005/2006) | CDC HRQoL-4 scale | Sleep characteristics | In-home interview-administered questionnaire using computer |
| Helmick et al(19) | 2014 | Americas | US | Cross-sectional | 10,676 | 20-59 | 8 (good) | National Health and Nutrition Examination Survey - NHANES (2003-2006) e NHANES (2009-2010) | CDC HRQoL–14 | Skin conditions (psoriasis) | In-home interviewing, with interviewer-administered questionnaire |
| Der-Martirosian et al(21) | 2013 | Americas | US | Cross-sectional | 1,379* | >65 | 8 (good) | National Survey of Women Veterans (NSWV) | SF-12 | Health in general and access to healthcare | Computer-assisted telephone interviewing, with interviewer-administered questionnaire |
| Biddle et al(22) | 2009 | Americas | US | Cross-sectional | 1,189* | 30-70 | 7 (fair) | Nationwide Survey of Female Sexual Health (2004-2005) | SF-12  EQ-5D | Sexual dysfunction during menopause | In-home interviewing, with interviewer-administered questionnaire |
| Stewart et al(23) | 2008 | Americas | US | Cross-sectional | 35,200 | Adultos | 6 (fair) | Medical Expenditure Panel Survey (MEPS 2000/2002) | SF-12  EQ-5D | Self-reported overall health | Self-administered email questionnaire |
| Gallegos-Carrillo et al(24) | 2009 | Americas | Mexico | Cross-sectional | 2,788 | >60 | 10 (good) | “Integral study of depression among older adults in Mexico City’s Mexican Institute of Social Security (IMSS) Policyholders” | SF-36 | Quality of life and social relationships | In-home interviewing, with interviewer-administered questionnaire |
| Kim et al(52) | 2018 | Asia | Korea | Cross-sectional | 2,349 | >70 | 9 (good) | Korea National Health and Nutritional Examination Survey (KNHANES 2008-2011) | EQ-5D | Chronic diseases, life style, weight loss | Interview in a mobile unit with self-administered questionnaire |
| Park et al(53) | 2017 | Asia | Korea | Cross-sectional | 8,976 | >50 | 8 (good) | Korea National Health and Nutrition Examination Survey (KNHANES 2010-2012) | EQ-5D | Joint diseases (osteoarthritis of the knee) ) | Interview in a mobile unit with self-administered questionnaire |
| Chung & Han(59) | 2017 | Asia | Korea | Cross-sectional | 229,131 | >19 | 7 (fair) | Korean Community Health Survey (KCHS 2010) | EQ-5D  EQ-VAS | Respiratory diseases (asthma) | In-home interviewing, with self-administered questionnaire |
| Hong et al(60) | 2016 | Asia | Korea | Cross-sectional | 8,963 | >50 | 9 (good) | Korean National Health and Nutrition Examination Survey (KNHANES-2010-2012) | EQ-5D  EQ-VAS | Joint diseases (osteoarthritis of the spine ) | Interview in a mobile unit with self-administered questionnaire |
| Park & Park(61) | 2016 | Asia | Korea | Cross-sectional | 28,382 | >19 | 9 (good) | Korean National Health and Nutrition Examination Survey (KNHANES) 2008-2012) | EQ-5D | Chronic diseases | Interview in a mobile unit with self-administered questionnaire |
| Kim et al(62) | 2015 | Asia | Korea | Cross-sectional | 2,165 | >50 | 8 (good) | Korean National Health and Nutrition Examination Survey (KNHANES 2010-2011) | EQ-5D  EQ-VAS | Chronic diseases | Interview in a mobile unit with self-administered questionnaire |
| Kim et al(63) | 2015 | Asia | Korea | Cross-sectional | 2,953 | >50 | 6 (fair) | Korea National Health and Nutrition Examination Survey (KNHANES 2010) | EQ-5D  EQ-VAS | Joint diseases and back pain | Interview in a mobile unit with self-administered questionnaire |
| Kim & Cho(64) | 2014 | Asia | Korea | Cross-sectional | 7,839 | 19-65 | 5 (fair) | Korea National Health and Nutrition Examination Survey KNHANES (2007-2009) and KNHANES (2010-2012) | EQ-5D | Factors associated with smoking cessation | Interview in a mobile unit with self-administered questionnaire |
| Sanson-Fisher et al(65) | 2013 | Asia | Korea | Cross-sectional | 897 patient and carer pairs | >18 | 9 (good) | The Korean National Cancer Center and nine other regional cancer centers. | CQoLC-K  EORTC-QLQ-C30 | Mental health of carers | In-home interviewing, with self-administered questionnaire |
| Vietri et al(66) | 2015 | Asia | Japan | Cross-sectional | 352 | >18 | 9 (good) | Japan National Health and Wellness Survey (NHWS 2010 and NHWS 2011) | SF-12 | Mental health | Online panel self-administered questionnaire. |
| Nishimura et al(54) | 2014 | Asia | Japan | Cross-sectional | 2,564 | Adultos e older persons | 8 (good) | J-ASPECT study group (2011) | SF-36 | Health in general | Self-administered mail questionnaire |
| Liu et al(55) | 2012 | Asia | Japan | Case-control | 37,683 | >18 | 7 (fair) | Japan National Health and Wellness Surveys (NHWS) | SF-12 | Infectious diseases  (Hepatitis) | Self-administered email questionnaire |
| Cho et al(56) | 2009 | Asia | Japan | Cross-sectional | 2,509 | 18-64 | 5 (fair) | Korean Epidemiological Catchment Area (KECA-R 2006-2007) | EQ-5D | Mental health | In-home interviewing, with self-administered questionnaire |
| Jankhotkaew et al(57) | 2017 | Asia | Laos and Thailand | Cross-sectional | 2,783 | 18-64 | 7 (fair) | WHO-ThaiHealth  (2012-2013) | EQ-5D | Impact on quality of life of people living with people with heavy drinkers | In-home interviewing, with interviewer-administered questionnaire |
| Huang et al(58) | 2010 | Asia | Thailand | Cross-sectional | 15,340 | 18-64 | 9 (good) | National Health Interview Survey in Taiwan (2005) | SF-36 | Food consumption,  obesity | In-home interviewing; who filled in the questionnaire not reported. |
| Langenbruch et al(32) | 2016 | Europe | Germany | Cross-sectional | 1,265 | >18 | 7 (fair) | PSO Health3 | FLQA-d  DLQI  EQ-VAS | Skin conditions | Interviews in health centers with self-administered questionnaire |
| Schunk et al(33) | 2015 | Europe | Germany | Cross-sectional | 846 | 45-74 | 9 (good) | KORA S4 (1999-2001);  SHIP 0 (2000-2003); DHS (2003-2004); CARLA (2002-2006); GNHIES 98(1997-1999) | SF-36 (GNHIES98, DHS)  SF-12 (KORA, SHIP e CARLA) | Chronic diseases  (Type II diabetes) | Interviews in study centers with self-administered questionnaire, except in KORA (face-to-face interview). |
| Langenbruch(44) | 2012 | Europe | Germany | Cross-sectional | 2,449 | >18 | 7 (fair) | PSO Health 1 and 2 | DLQI  EQ-VAS | Skin conditions | Self-administered mail questionnaire |
| Madsen et al(45) | 2015 | Europe | Denmark | Cross-sectional | 1,447 | >18 | 6 (fair) | Survey conducted by the University of Copenhagen, Denmark | EORTC QLQ-C30 | Cancer | Self-administered mail questionnaire |
| Peuckmann et al(46) | 2009 | Europe | Denmark | Cross-sectional | 1,316* | >40 | 8 (good) | Danish Breast Cancer Cooperative Group (DBCG) 2004-2005 | SF-36 | Pain and other sequelae cancer survivors | Interviews in health centers with self-administered questionnaire |
| Peuckmann et al(47) | 2009 | Europe | Denmark | Cross-sectional | 1,316 | >40 | 8 (good) | Danish Breast Cancer Cooperative Group (DBCG) 2004-2005 (taken from this database) | SF-36 | Characteristics of cancer survivors | Interviews in health centers with self-administered questionnaire |
| Fernandez-Mayoralas et al(48) | 2012 | Europe | Spain | Cross-sectional | 1,106 | >60 | 7 (fair) | CadeViMa-Spain (2008) | EQ-5D  EQ-VAS | QoL in community-dwelling older adults | In-home interviewing, with interviewer-administered questionnaire |
| Williet et al(49) | 2017 | Europe | France | Cross-sectional | 1,185 | >18 | 8 (good) | Nationwide survey of members of the French patients’ society Association Francois Aupetit [AFA] | SIBDQ  SF-36 | Intestinal diseases (inflammatory bowel disease ) | Self-administered mail questionnaire |
| Audureau et al(50) | 2016 | Europe | France | Cross-sectional | 21,239 | 25-64 | 8 (good) | French Decennial Health Survey (2003) | SF-36 | Obesity and comorbidities | In-home interviewing, with self-administered questionnaire |
| Douab et al(51) | 2014 | Europe | France | Cross-sectional | 4,339 | >18 | 8 (good) | VESPA ANRS-EN12 (2002-2003) and ANRS-VESPA2 (2011-2012) | SF-36  (VESPA -2002/2003)  SF-12  (VESPA 2 - 2011/2012) | HIV/AIDS | Interview in hospital with self-administered questionnaire |
| Gentile et al(34) | 2013 | Europe | France | Cross-sectional | 1,061 | >18 | 7 (fair) | Data from 8 regions of France, participants of the French Renal Epidemiology and Information Network and CRISTAL database | SF-36  RTQ (ReTransQoL) | Renal transplant recipients | Self-administered mail questionnaire |
| Hoopman et al(35) | 2009 | Europe | Netherlands | Cross-sectional | 10,414 | >18 | 8 (good) | Second Dutch National Survey of General Practice | SF-36 | Comparison between minorities and ethnic groups in the Netherlands | In-home interviewing with interviewer-administered questionnaire. |
| Upton et al(36) | 2016 | Europe | England | Cross-sectional | 658 | 18-74 | 5 (fair) | Health Survey for England - HSE (2010) | AQLQ-M | Respiratory diseases (Asthma) | Interviewer-administered telephone questionnaire |
| Thomas et al(37) | 2015 | Europe | England | Cross-sectional | 195,364 | >18 | 6 (fair) | English General Practice Patient Survey (GPPS 2011-2012) | EQ-5D | QoL in informal carers | Mail/on line questionnaire or interviewer-administered telephone questionnaire |
| Mishra et al(38) | 2014 | Europe | England | Cohort | 2,292 | 60-64 | 7 (fair) | National Survey of Health and Development- NSHD | SF-36 | Childhood and maternal effects on physical health related quality of life five decades later | Self-administered mail questionnaire |
| Anokye et al.(39) | 2012 | Europe | England | Cross-sectional | 5,537 | 40-60 | 7 (fair) | Health Survey for England - HSE (2008) | EQ-5D | Physical activity | In-home interviewing, with self-administered questionnaire |
| Masood et al(40) | 2017 | Europe | United Kingdom | Cross-sectional | 1,277 | >65 | 7 (fair) | Adult Dental Health Survey (ADHS, 2009) | OHIP-14 | Oral health | In-home interviewing; who filled in the questionnaire not reported. |
| Mujica-Mota et al(41) | 2015 | Europe | United Kingdom | Cross-sectional | 831,537 | >18 | 9 (good) | English General Practice Patient Survey (GPPS, 2011–2012). | EQ-5D | Morbidity and multi-morbidity | Mail questionnaire, Self-administered mail/online questionnaire or telephone questionnaire |
| Lindberg et al(42) | 2011 | Europe | Sweden | Cross-sectional | 4,875 | 18-84 | 6 (fair) | The Swedish Survey of Living Conditions | SF-36 | Skin conditions (eczema) | Self-administered mail questionnaire |
| Luthy et al(43) | 2015 | Europe | Switzerland | Cross-sectional | 2,888 | >65 | 8 (good) | ‘‘Vivre/Leben/Vivere’’(2011) | EQ-5D | QoL of community-dwelling elderly people | In-home interviewing, with self-administered questionnaire |
| Neil et al(67) | 2018 | Oceania | Australia | Cross-sectional | 1,163 | 18-64 | 8 (good) | Australian National Survey of Psychosis (2010) | AQoL-4D | Mental health (psychotic illness) | Interview in public health services with interviewer-administered questionnaire |
| Crocombe et al(68) | 2013 | Oceania | Australia | Cross-sectional | 3,730 | 18-65 | 10 (good) | Defense Deployed Solomon Islands- (SI)  Health Study and the National Survey of Adult Oral Health (NSAOH, 2004–2006) | OHIP-14 | Oral health | Interviewer-administered telephone questionnaire |
| Parslow et al(69) | 2011 | Oceania | Australia | Cross-sectional | 2,286 | >60 | 6 (fair) | II National Survey of Mental Health and Well-Being (2007) | SF-12  (one general question about health) | Development and testing of a multidimensional QoL model using data from a sample of older Australians | In-home interviewing with self-administered questionnaire. |

* Studies only with women.
